# 38. DO NMDAR ANTIBODIES CAUSE SCHIZOPHRENIA?

**DOI:** 10.1093/schbul/sby014.153

**Published:** 2018-04-01

**Authors:** Belinda Lennox

**Affiliations:** University of Oxford

## Abstract

**Overall Abstract:** NMDAR antibodies have been described in association with some people with schizophrenia. However the finding is still controversial, and in particular some groups describe equal prevalence of antibodies in patients with schizophrenia as other disease controls, or in healthy control subjects. this symposium includes the leading academics undertaking research in this area and will discuss the hot topics in the area, reviewing the latest evidence from a range of perspectives. This will include comparison of testing methods for NMDAR antibodies, discussion of functional effects of NMDAR antibodies with relevance to schizophrenia, an update on prevalence studies of antibodies in psychosis and at risk mental states, and clinical data on the experience of screening patients for NMDAR antibodies in psychiatric hospitals. The discussant is Sarosh Irani, associate professor in neurology at the University of Oxford, who led the first European case series description of NMDAR antibodies.

